# Association of Viewing the Films *Joker* or *Terminator: Dark Fate *With Prejudice Toward Individuals With Mental Illness

**DOI:** 10.1001/jamanetworkopen.2020.3423

**Published:** 2020-04-24

**Authors:** Damian Scarf, Hannah Zimmerman, Taylor Winter, Hannah Boden, Sarah Graham, Benjamin C. Riordan, John A. Hunter

**Affiliations:** 1 Department of Psychology, University of Otago, Dunedin, New Zealand; 2 Department of Psychology, Victoria University of Wellington, Wellington, New Zealand; 3 Discipline of Addiction Medicine, Central Clinical School, Faculty of Medicine and Health, The University of Sydney, Sydney, New South Wales, Australia

## Abstract

This survey study assesses the association of viewing the film *Joker *with level of prejudice toward individuals with mental illness.

## Introduction

The movie *Joker* provides an origin story for its namesake character, played by award-winning actor Joaquin Phoenix. Phoenix’s character Arthur is depicted as having a mental illness; he visits Arkham State Hospital to receive medication and frequently displays symptoms that suggest some form of serious mental illness. Because of budget cuts, Arthur stops receiving his medication and consequently carries out a campaign of violence.

*Joker* is the first R-rated film to earn more than $1 billion at the box office, with more than 100 million people viewing it worldwide. Because *Joker* continues the tradition of movies depicting individuals with mental illness as violent,^1,2^ it has reignited discussion about the role of media in perpetuating prejudice toward those with a mental illness.^3^ To investigate the associations of the *Joker *with prejudice toward those with mental illness, we had a community sample attend a screening of *Joker* or *Terminator: Dark Fate* (as the control condition) and complete a measure of mental illness prejudice before and after watching their assigned movie. We hypothesized that, compared with viewing *Terminator: Dark Fate*, viewing *Joker* would be associated with higher levels of prejudice toward individuals with mental illness.

## Methods

In this survey study, participants enrolled from November 22, 2019, to December 4, 2019. On December 5, 2019, participants attended a movie theater in Dunedin, New Zealand, and were randomly assigned to watch either *Joker* or *Terminator: Dark Fate*. The only inclusion criterion was that participants be aged 18 years or older. Participants completed an online consent form before being presented with the premovie survey. Participants completed the 28-item Prejudice Toward People With Mental Illness (PPMI) scale^4^ before and after watching their assigned movie. An overall association of viewing either movie with prejudice was estimated using confirmatory factor analysis in R version 3.6.1 (R Project for Statistical Computing), with the *lavaan *command.^5^ Change in prejudice was assessed using a lagged Bayesian regression, using a weakly informative normal prior with a mean of 0 and an SD of 1. Age, sex, history of mental illness, and movie were included as covariates. Association was determined based on a posterior probability greater than 95%. Posterior probability is the percentage of posterior samples above or below 0, depending on which direction has the highest probability (akin to a 1-tailed test). The study was reviewed and approved by the University of Otago Human Ethics Committee.

## Results

Overall, 164 individuals participated in the study, approximately evenly split between viewing *Joker* (84 [51.2%]) and *Terminator: Dark Fate* (80 [48.8%]). Samples were similar in terms of age, sex, and race/ethnicity (Table 1). Participants viewing *Joker* had a mean (SD) PPMI of 2.99 (0.66) before the movie and 3.20 (0.78) after the movie. Participants viewing *Terminator: Dark Fate* had a mean (SD) PPMI score of 2.91 (0.61) before the movie and 2.88 (0.70) after the movie (Table 1). The lagged Bayesian regression revealed neither age nor sex was associated with PPMI scores. Whether participants have had or currently have a mental illness was associated with lower PPMI scores (estimate, −0.21 SD; 95% CI, −0.4 to 0 SD; posterior probability, 100.0%). Consistent with our hypothesis, viewing *Joker* was associated with a 0.37 (95% CI, 0.19 to 0.55) SD increase in PPMI score (posterior probability, 100.0%) (Table 2).

**Table 1.** Sample Characteristics

Characteristic	No. (%)		
	Individuals viewing *Joker *(n = 84)	Individuals viewing *Terminator: Dark Fate* (n = 80)	Total (N = 164)
Women	53 (63.1)	49 (61.3)	102 (62.2)
Age, mean (SD), y	30.3 (10.5)	29.9 (9.8)	30.1 (10.1)
Race/ethnicity			
White	51 (60.7)	31 (38.8)	82 (50.0)
Asian	23 (27.4)	26 (32.5)	49 (29.9)
Maori or Pacifica	10 (11.9)	12 (15.0)	22 (13.4)
Other	9 (10.7)	11 (13.8)	20 (12.2)
PPMI scale score, mean (SD)^a^			
Before the movie	2.99 (0.66)	2.91 (0.61)	2.95 (0.63)
After the movie	3.20 (0.78)	2.88 (0.70)	3.04 (0.75)

Abbreviation: PPMI, Prejudice Toward People With Mental Illness.

^a^ Score on PPMI in this table was presented as the mean scale response; however, a standardized latent variable was used in regression analysis.

**Table 2.** Results of Lagged Bayesian Regression Analysis on Change in Prejudice After Watching *Joker*

Covariate	Estimate (95% CI)	PP, %^a^
Intercept	0.08 (−0.1 to 0.3)	83.6
Prejudice, lag	0.91 (0.8 to 1.0)	100.0
Age	0.02 (−0.1 to 0.1)	74.1
Female sex	0.00 (−0.2 to 0.2)	50.2
Mental health history	−0.21 (−0.4 to 0)	100.0
Viewing *Joker*	0.37 (0.2 to 0.6)	100.0

Abbreviation: PP, posterior probability.

^a^ Posterior probability is the probability an effect deviates from 0 in its given direction, eg, a coefficient of −0.21 and posterior probability of 100.0% means all posterior samples were less than 0.

## Discussion

*Joker* was associated with higher levels of prejudice toward those with mental illness. Beyond prejudice, associating mental illness with violence may erode support for policies that we know to be beneficial for those with mental illness (eg, integration into communities). Additionally, *Joker* may exacerbate self-stigma for those with a mental illness, leading to delays in help seeking.^6^ A limitation of the current study is that we did not assess whether viewing *Joker* was associated with actual behavior.

In *The Dark Knight*, Joker asks, “Why so serious?” One might level that question at us, arguing that *Joker* is nothing to be concerned with. However, what this view ignores is the profound consequences prejudice has on those with a mental illness.
